# Determinants of antiretroviral therapy adherence in northern Tanzania: a comprehensive picture from the patient perspective

**DOI:** 10.1186/1471-2458-12-716

**Published:** 2012-08-30

**Authors:** Ramsey A Lyimo, Marijn de Bruin, Jossy van den Boogaard, Harm J Hospers, André van der Ven, Declare Mushi

**Affiliations:** 1 Kilimanjaro Clinical Research Institute, Kilimanjaro Christian Medical Centre, P.O.Box 2236, Moshi, Tanzania; 2 Department of Communication Science, Wageningen University, Wageningen, The Netherlands; 3 Department of General Internal Medicine, Radboud University Nijmegen Medical Centre, P.O. Box 9101, Nijmegen, 6500 HB, The Netherlands; 4 Faculty of Psychology and Neuroscience, Maastricht University, P.O. Box 616, Maastricht, 6200 MD, The Netherlands; 5 Community Health Department, Kilimanjaro Christian Medical College, P.O.Box 2240, Moshi, Tanzania

## Abstract

**Background:**

To design effective, tailored interventions to support antiretroviral therapy (ART) adherence, a thorough understanding of the barriers and facilitators of ART adherence is required. Factors at the individual and interpersonal level, ART treatment characteristics and health care factors have been proposed as important adherence determinants.

**Methods:**

To identify the most relevant determinants of adherence in northern Tanzania, in-depth interviews were carried out with 61 treatment-experienced patients from four different clinics. The interviews were ad-verbatim transcribed and recurrent themes were coded.

**Results:**

Coding results showed that the majority of patients had basic understanding of adherence, but also revealed misconceptions about taking medication after alcohol use. Adherence motivating beliefs were the perception of improved health and the desire to live like others, as well as the desire to be a good parent. A de-motivating belief was that stopping ART after being prayed for was an act of faith. Facilitators of adherence were support from friends and family, and assistance of home based care (HBC) providers. Important barriers to ART adherence were the use of alcohol, unavailability of food, stigma and disclosure concerns, and the clinics dispensing too few pills. Strategies recommended by the patients to improve adherence included better Care and Treatment Centre (CTC) services, recruitment of patients to become Home Based Care ( HBC) providers, and addressing the problem of stigma through education.

**Conclusion:**

This study underscores the importance of designing tailored, patient-centered adherence interventions to address challenges at the patient, family, community and health care level.

## Background

The availability and use of antiretroviral therapy (ART) has increased considerably in Sub-Saharan Africa in recent years. In Tanzania, 1.4 million people (5.7% of the population aged 15–49 years) were living with HIV/AIDS at the end of 2009. In the same year, 55.2% of the 425,725 Tanzanian children and adults in need of ART were receiving it [1,2]. The clinical targets of ART have been defined as full repression of viral multiplication, restoration of the immune response, arresting the progression of disease, increasing survival rates, reducing morbidity and enhancing the quality of life [3]. Acquisition of the full benefits of ART depend on continuously high adherence to the medication (i.e., >90-95% correct intake of prescribed doses) [4]. However, many patients taking ART do not manage to achieve such high levels of adherence [5].

To design effective adherence promoting interventions that are sensitive to the local context, culture and facilities, there is a need for a thorough understanding of the relevant, modifiable behavioral determinants. Established theories on health behavior suggest that the first possible determinant is patients’ knowledge on adherence to ART, based upon which people form their treatment outcome expectancies [6]. Moreover, it is expedient to examine motivational factors to adherence, which according to the Theory of Planned Behavior (TPB) [7] comprise an attitudinal dimension (rational and affective), a social dimension (i.e., the belief that one is being supported or hindered in executing the behavior by important others), as well as the patient’s confidence in his or her ability to adhere to the required standards. These 3 sources of motivation determine people’s behavioral intentions (e.g., ‘I want to take these pills every day’). However, the degree to which patients are successful in translating their adherence intentions into behavior may depend on experienced obstacles, facilitators, and patients’ self-regulation skills to overcome these problems [8-10].The individual-level adherence determinants can be influenced by the patient’s interpersonal environment (e.g., practical support, stigma), the medication (e.g., side effects, complexity), and the health care provided (e.g., the quality of adherence support at the clinic, privacy at the clinic) [11-13]. Hence, adherence determinant studies should try to understand patients’ emotions, cognitions and behaviors within their specific context.

Although multiple studies on determinants of ART adherence have been conducted in SSA, the continent is large and consists of many different countries with their own history, culture, health care system, infrastructure, and so forth; factors that may present a unique mix of challenges and resources for patients treated for HIV, which are also likely to change over time. Despite studies on determinants of ART adherence conducted in Tanzania, still there is a need for through and updated understanding of adherence barriers and facilitators in order to provide high-quality adherence support to secure long-term treatment effectiveness and prevent resistance development among HIV patients in Tanzania. This study aimed to provide that understanding by combining the virtues of qualitative research (e.g., detailed information on particular beliefs and barriers, taking context into account) with representativeness of the findings by including a substantial number of patients from 4 different HIV clinics.

## Methods

### Study site and participants

This qualitative study was conducted with patients in Care and Treatment Centre (CTC) clinics located in three rural and one urban hospital of the Tanzania Kilimanjaro Region. Two of the four CTC clinics operate in hospitals owned by religious institutions (Roman Catholic Church and Evangelical Lutheran Church of Tanzania) while the other two clinics are government-owned. All clinics offer care to the general public, regardless of religious background (note that the great majority, more than 95%, of the people are Christian). The number of patients using ART in the included clinics range from 500–1500 per clinic. The native populations of the study area are Chagga. People’s main occupation is agriculture, which comprise of subsistence coffee, maize, beans and banana farming as well as animal keeping.

Adult patients using ART for at least 6 months and who were regular clients of the CTC clinic (rather than temporary visitors) were eligible to participate. Patients were approached during routine clinic visits from February 2010 to March 2010. The researchers were introduced to eligible patients by the CTC staff and explained that the aim of the study was to explore the experiences of the patients in their medication use, their perceptions about facilitators and barriers of adherence, and how being adherent could be facilitated. Patients were informed that participation in the study was voluntary and that refusal would not affect their relationship with their health care provider. A total of 61 patients were conveniently selected to participate in the study from the four CTC clinics. The study patients were approached on their clinic appointment day and asked to participate in the study. All patients who agreed to participate provided a signed consent. The research was approved by the Tanzania National Institute for Medical Research (NIMR).

### Data collection methods and tools

The research team consisted of two experienced researchers and three trained research assistants who were not part of the community where the study was conducted. This was intentionally done to avoid patients feeling uncomfortable to discuss their disease and the challenges they faced, and avoid inducing fear of HIV status disclosure. All researchers were trained on how to systematically conduct the interview. They were also trained to first create an open atmosphere and ensure patients that all information would be kept confidential and anonymous. The interviews were pilot -tested in a clinic where data was not collected. The interview questions were guided by key-behavioral determinants explained in the introduction. Hence the interview guide consisted of topics addressing: 1. Patient’s knowledge about adherence, 2. Motivational factors (attitudes, emotions, norms of important others, and perceived behavioral control), 3. Barriers and facilitators of adherence (individual, interpersonal, treatment or health care factors) and 4. Strategies for improving adherence. Since this was a semi-structured interview, the interview guide was used as a tool to help interviewers ask questions in a flexible way and to follow up on previously identified themes. All interviews were conducted in Kiswahili (Tanzania’s national language), in a quiet place in the hospital. The interviews were recorded using digital recorders and transcribed verbatim. The transcribed text from each participant was independently translated by two researchers from Kiswahili to English.

### Data analysis

Data from the interviews were analyzed manually using content framework analysis [14]. The procedure involved identifying and coding the data into respective topics followed by interpretation. All five interviewers separately identified themes and later agreed on common themes. The thematic framework was identified by relating back to the theoretical framework original study aims and objectives and incorporating emerging issues and concepts. The framework was used to develop themes and categories of themes. Interpretation concerned defining concepts, mapping array and nature of the phenomena, and categorizing responses that had similar qualities or characteristics.

## Results

Sixty-one patients (36 female and 25 male) took part in the study. Their mean age was 34 years (range: 18–60 years). Nearly half (44%) of the participants were married, 20% widowed, while the proportion of single and divorced patients was 18% for both. The majority of participants (62%) had completed primary education, 16% had some level of secondary education, and 18% had few years of primary education or no education. The socio-demographic and treatment characteristics of the study participants are shown in Table 1.

**Table 1:** Socio-demographic& disease characteristics of participants

**Characteristics**	**Number (n = 61)**	**%**
*Gender*		
Female	36	59.1
Male	25	40.9
*Age*		
18–29	3	4.9
30-49-	43	70.5
50+	11	18.0
Missing	4	6.6
Marital Status		
Single	11	18.0
Married	27	44.3
Divorced	11	18.0
Widow	12	19.7
Education		
No education	3	4.9
Incomplete primary	8	13.1
Standard seven	38	62.3
Secondary	10	16.4
College/University	2	3.3
Year Started ARV		
1999-2001	4	6.6
2002-2004	8	13.1
2005-2007	22	36.0
2008-2009	27	44.3
HIV Disclosure		
Disclosed	53	86.9
Not disclosed	8	13.1

### Understanding the concept of adherence

With regard to patients’ knowledge about ART adherence, all participants demonstrated to understand that they are supposed to take the pills at the right time and lifelong. Common statements regarding adherence to ART were “*To take pills as instructed”, “To observe time in taking pills*” and “*To take pills without stop*”. However, patients also had misconceptions about adherence, which were mainly related to alcohol consumption. Patients indicated that the use of alcohol was strictly prohibited to patients on ART, or that ART should not be taken after alcohol had been consumed because then it would not work anymore. For example, 42 years-old-man said *“We have been told that this medicine does not work well with alcohol*” and another man aged 39 years said *“I sometimes stop the medicine because; once you start using this pill it is not allowed to drink alcohol”.*

### Beliefs that motivate or de-motivate ART adherence

When asked to explain what motivates them to continue with ART, nearly two-third (60%) of respondents gave reasons such as: improved health after starting ART, the desire to live and take care of children, wanting to live like others without HIV, and the desire of approval from health providers at the clinics. The following quotations illustrate these internal and external motivational drives: “*I am motivated because my health has improved a lot. I will never stop using the medication, maybe until when ARTs are out of stock or no longer produced*” (Female, 41 years); “*I don’t want to die. If I stop using ART I will die. I want to live longer in order to take care of my family*” (Female, 36 years); “*I have to adhere to the treatment because I want to live like an ordinary person*” (Male, 47 years);“*There was a time I missed my medication for one month; when I went to the hospital nurses were mad at me. I learned that if I don’t adhere next time I will be in trouble again*” (Male, 42 years).

On the other hand, religion contributed to a lower adherence motivation among some patients, since five patients indicated that they were discouraged from continuing taking their pills after being prayed for as an act of faith by some religious groups. For example a woman, 46 years said “*There is a religious group in our village who pray for us and ask us to stop taking the ART to demonstrate our faith in healing*”. Patients also mentioned that some patients stopped taking their pills after their health status had improved, because they felt well and did not perceive a pressing need to continue with the medication.

### Barriers to ART adherence at the individual level

Alcohol use was not only a reason for intentional non-adherence due to not understanding how to use the medications in relation to alcohol consumption, it was also reported as causing lack of consistency in taking medication when drinking alcohol. For example, a woman aged 30 years said: “*My husband does not have regular time to take the medication because he drinks alcohol a lot. He can take pills even at midnight sometimes if he is drunk he doesn’t take the pills until next day.*” Another patient (Male, 60 years) responded when asked what he perceived to be a barrier to adherence said *“Binge drinking is a big problem”.* Inaccessibility of sufficient food was also mentioned as one of the barriers to ART adherence. A female of 47 years said: “*If you don’t have enough food it is difficult to take the medicines; these medicines are very strong so you must eat well*”. Another male patient said: “*It is hard to adhere to medication because after taking the medicines I need to eat and if you don’t have food you decide to stop the medicine*”.

### Barriers and facilitators at the interpersonal and community level

Stigma and disclosure concerns were identified as potential barriers to ART adherence. It was noted that nearly a quarter (23%) of patients avoid going to a nearby CTC clinic due to the fear that fellow community members may get to know their positive HIV status. In other words, they choose to enroll in a CTC clinic further away from their homes to avoid being identified by their fellow community members. Moreover, some feared to disclose their status due to the fear of being stigmatized. For example, a 49-years-old male said: “*No one knows that I am under ART, I can’t tell my wife or relatives. They will tell everybody”*. It was also noted that some patients avoid disclosing their positive HIV status to their parents and loved ones because they do not want to hurt them. A male participant of 44 years said: “*My parents will feel very bad if they find out about my positive HIV status”*. Another female of 45 years said: “*I can’t tell my parents. They have high blood pressure; I don’t want them to be in trouble*”. Yet another participant (male, 49 years) said, *“I have disclosed to my young brother, my wife and few relatives, who understand me. But I have not told my parents, they can die”.*

On the other hand, disclosure of HIV positivity was perceived as a facilitator of adherence. We noted that 87% of the study patients had disclosed their HIV status to other people, particularly to family members or HIV positive friends. Five respondents said that everyone in the community knows their status because they are peer educators and members of the People Living with HIV/AIDS (PLWHA) group. From the interviews we also noted that those who had disclosed their HIV status, especially to family members, received support like assistance in household chores during sickness. Moreover, the people to whom they had disclosed their HIV status reminded them about pill taking. Children were mentioned as the most important group of people in reminding parents about their medication. For example, a 40-year-old woman stated *“My children are very supportive especially in reminding me about pill taking*”, yet a woman aged 60 years added, “*My children remind me*” and a man aged 33 years said “*My wife and few relatives whom I disclosed to, remind me*

### Barriers and facilitators at the treatment and health care level

When asked to share their experiences on taking ART, only six patients said they did not face difficulties in taking the medication while the others indicated to have had some difficulties, particularly related to the occurrence of side effects such as feeling sleepy or hungry, having swollen legs, neuropathy, diarrhea and joint pains: *“When I started my ART, I experienced headaches and vomited most of the times. But nowadays I don’t feel that problem anymore.”* (Male, 50 years). Out of 55 patients who experienced side effects, 31 (56%) said the side effects lasted for a short period, whereas 20 (33%) patients experienced severe side effects and had to change to another type of medication. However, patients did not indicate that these side effects influenced their adherence.

Regarding the care provided, six participants reported that there are times the CTC clinics run short of pills. In such cases patients either do not get medication at all, or they are given an insufficient number of pills. In addition, some respondents said “*Sometimes doctors make mistakes: they give a next appointment date that does not correspond with the number of pills dispensed*”. For example a female patient aged 47 years said: *“It is common to finish drugs before the appointment date. This month I missed pills for two days before my appointment day was due.*” Another observation was that because CTC clinics sometimes dispense too few pills, some patients share their pills with a friend or spouse when one runs short of medication. For example, a 46-years-old woman said: “*I have a neighbor whom we share medicines, if one runs short of drugs*”.

Regarding the organization of the ART clinic, some patients indicated dissatisfaction about the set-up of the clinic. For example, a male patient aged 37 years remarked: “*Separation of the CTC clinic from the other department buildings of the hospital is a problem because when one goes to collect the ARVs, there is fear that other people will know our problem”.*

Finally, the active role of trained home based care (HBC) providers who bring medication to patients at their homes in case of illness, were indicated by the majority of patients as being an important facilitator of adherence.

### Patients’ suggestions for strategies to improve adherence

As regards to measures to be taken to improve ART adherence, the majority of patients recommended firstly the improvement of services at the CTC clinics, and particularly the availability of pills at the clinics at all times. Furthermore, patients recommended the involvement of PLWHA to be recruited as HBC providers because they felt that they would be more acceptable to patients. They also said it would be helpful to have more social support from family members such as reminding them to take medication, supporting them with food and helping them in house hold chores on occasion of illness. Finally, and linked to the perceived importance of social support, the majority of patients indicated that addressing the problem of stigma through provision of appropriate education to patients as well as to communities would improve ART adherence as the following quotes from patients indicate “*I would like the surrounding community to be educated and understand the disease and stop stigmatizing and, help me with food”.*(Male, 41 years). Another female aged 42 years responded “*Community members should be educated not to stigmatize me but cooperate with me”*

## Discussion

Poor adherence to ART is a well-known problem with implications for patients’ and public health due to the spread of (resistant) HIV [15]. This study sought to identify the most relevant determinants of adherence through interviews with 61 patients in 4 different health clinics in northern Tanzania anno 2010, six years after the introduction of ART in the region. Our findings indicate that the majority of patients have a basic understanding of the meaning of adherence to medication. The perception of improved health, the desire to live a similar life as non-infected community members, having children and being a good parent were identified as key-adherence motivating factors. Support from friends and family members and the active role of home based care providers were important facilitators of adherence. The use of alcohol, the unavailability of food, stigma, status disclosure concerns, and the clinics dispensing too few pills were identified as potentially important barriers to adherence. These findings suggest avenues for supporting adherence through changes at the individual, interpersonal, community and health care level.

According to behavioral theories, patients’ knowledge about a health behavior forms the basis for their so-called outcome expectancies, and the value attached to these outcomes which shapes people’s attitudes towards the behavior [7,16]. In the present study, the majority of patients had adequate basic understanding on adherence in the sense that they are supposed to take pills at the right time and lifelong. However, there was also what we perceived to be a common misunderstanding concerning taking medication and the use of alcohol. Some patients stop taking medication because they believe that one should not drink *at all* (not even incidentally and moderately) while on ARV. Hence, since alcohol drinking is part of culture in the study area, many patients are faced with dilemma to either stop drinking alcohol completely and take medication, or stop medication even when they drink a moderate amount of alcohol on a particular occasion. Similar beliefs have also been observed in a study by Sankar (2007), who found that 85% of his African-American respondents strongly believed that ART and alcohol do not go together [17]. What our study identified was that this belief about alcohol use seems to be, at least in this region, mainly introduced by the health care providers. No doubt their intention was to limit problematic alcohol (i.e., upon inquiry health care providers reported to do this for general health, promotion of safe sex, and to tackle the problem of excessive alcohol use while on ART) while keeping patients on ART, rather than patients not taking the medication when drinking (some) alcohol. However, the practice of prohibiting alcohol use completely when or ARV’s, which for many patients may be unrealistic, seems counterproductive and it is also not required from a pharmacological viewpoint. Hence, this finding suggest that in addition to patients with problematic drinking behavior, health care providers should be the focus of intervention efforts since they can better acknowledge that patients *will* consume alcohol, and instead of prohibiting alcohol discuss with patients under what conditions alcohol use is acceptable.

Knowledge and motivations are not only based on information received from others, but also arises from experiences patients have had with the treatment [9]. With regard to this aspect, patients mentioned improved health after the start of ART as a motivation to continue with ART. However, this same experience also turned out to de-motivate some other patients, since they stopped the use of ART after their health had improved. The latter perception has also been observed in other studies, where patients indicated difficulties in adhering during times of decreased symptoms [18,19]. The patients presumably felt less urgency to continue with the medication because the symptoms, which initially prompted them to take their medication, disappeared. It seems that to prevent this problem, it is important that health care providers intervene on the belief that medication is only required or beneficial when symptoms are present. Instead, as some patients indicated in this study, improved health is ideally perceived as a reason to continue the treatment because of its experienced benefits. Although the key difference between patients experiencing improved health as a reason to continue versus discontinue treatment were not identified in this study, a logical explanation seems to be the level of understanding or belief in how the disease and the treatment works i.e., patients’ explanatory model of the disease and treatment, cf., [20]. Indeed, although all patients knew the basic instructions for medication intake, very few patients in this study displayed understanding of how HIV works, affects the body and how the treatment disrupts that process. Hence, improved understanding of these (long-term) processes may prevent patients to halt treatment when symptoms disappear. Other key motivational beliefs were also identified, namely the need to take care of one’s family and the desire to live like an ordinary person. Identifying these key needs no an individual level, and linking them long-term adherence to these beliefs (in line with Motivation Interviewing and the Self-determination theory [21], seems to be important to induce high levels of motivation to adhere to ART.

Discrepancies between medical knowledge and patient beliefs were also observed in relation to the power of prayer, since some patients stopped using ART after they had been prayed for as an act of faith. Religion is an important part of life of the people in the study area and there are number of faith healers who claim to have the power to heal diseases including HIV/AIDS. Stopping treatment after prayer has also been observed in other studies [22-24] and it seems difficult to challenge such religious beliefs. A possible approach could be to mobilize church leaders, since they might be the most trustworthy source for patients to alter such beliefs (i.e., rather than physicians, nurses or community workers).

Furthermore, and in accordance with the theories discussed [7-10], the extent to which patients reported to be successful in adhering depended on whether their efforts were being facilitated or obstructed, and on their self-regulatory skills to cope with such barriers. Factors that facilitated adherence were at the interpersonal level (i.e., practical and emotional social support) [25], and at the health care level (i.e., home-based care providers). Factors that obstructed adherence were mentioned more often, and resided at the individual and interpersonal level (i.e., lack of food, fear for stigma and disclosure) as well as at the health care level (i.e., lack of privacy at the clinic, and insufficient availability or provision of pills). This form of malpractice, namely providing insufficient pills while stocks were sufficient, was also observed in a previous study by the authors in the same area [26]. After discussing this with clinics they changed their procedures and offered patients sufficient pills to bridge the time between two visits, including a few extra pills in case the patient could not make it to the clinic at the appointment date. Regarding the barriers at the individual and interpersonal level, these seem more difficult to alter. A lack of food common is common among people in this region. One recommended strategy is that health care providers discuss with patients facing this problem whether they can more strategically ration their food and otherwise take the medication in the absence of food (since first-line treatments in this region do not need to be combined with food). Another strategy is to attempt to reduce the financial burden for patients by lowering visit frequency to the clinic for adherent patients [27] or linking them with microcredit schemes [28]. Regarding the interpersonal level, successful, well-conducted stigma-reducing interventions are scarce although a recent review reveals there are several promising programs that can be adopted [29].

Side-effects were also reported by many patients as a problem, although the participants in the current study indicated that this did not affect their adherence, which is contrary to findings in many other studies in which side effects were found to hamper adherence e.g., [30,31]. This could be explained by the fact that patients in this area had an option to switch to another regimen, or simply by the fact that participants in this study were those patients still retained in treatment (i.e., those with severe side effects are more likely to drop out) [29]. Hence reported ways of dealing with barriers were changing medication by the health care provider, who should systematically inquire about such issues, and taking the medication until the side-effects resided.

Regarding strategies to improve adherence, our respondents recommended that the clinics should be integrated into the other services provided by the health care institution to maintain discretion. Some patients turned to clinics further away from their homes in order to avoid being recognized when visiting the clinic, which introduces additional barriers to the continued use of care due to the travel distance. Patients also indicated that clinics should provide sufficient pills and they stressed the importance of social support and the reduction of stigma about HIV in the community. Hence, patients perceived the key challenges to adherence to reside in their environment. Although these recommendations are highly valuable, the results of this study also suggest it is key to address behavioral determinants at the individual level: knowledge and misconceptions about the treatment and adherence; motivation to continue ART use despite waning of symptoms; skills and confidence for disclosure, coping with stigma, alcohol use and other barriers to taking ART continuously and life-long.

These findings and patients’ suggestions have broader implications for other HIV care in Tanzanian clinics as well as for future research. In sum, our recommendations for supporting adherence based on this study are, first, to improve counseling by the health care providers [c.f., [32]. This counseling should focus on providing patients a deeper understanding of how HIV and the treatment works and the responsible use of alcohol (knowledge); appealing to patients’ personally relevant goals achieved through adhering to the treatment (e.g., ability to take care of family) and reducing de-motivating factors (such as dealing with side-effects or promoting disclosure to deal increase a sens of support) [33]; and dealing with barriers such as a lack of food. Clinic changes recommended are to always provide patients with a more than sufficient amount of pills and to allow patients to keep their HIV status confidential by not creating a separate location for HIV treatment in the clinic. Finally, community support (or at least, a reduction of perceived stigma) and eradicating the belief that prayer can cure HIV could be tackled by combined efforts of community or governmental organizations and religious leaders. Hence, in order to improve adherence and quality of life of patients with HIV, a multi-level health promotion intervention is required [33,34]. However, which interventions are most effective and feasible in this setting -also considering the limited time health care professionals have per patient - should be subject to future intervention studies.

This study had several limitations. It is a qualitative study relying on patients self-reports and perception of adherence influences retrospectively. Moreover, socially desirable answers may have occurred. Finally, although there was a relatively large sample size and patients were selected from four clinics, the sample is too small for the results to be generalized to all Tanzanian patients and clinics.

## Conclusion

In conclusion, having HIV and adhering to ART remains to pose substantial challenges to patients in Tanzania. Adherence seems to be affected by multiple factors at the individual, interpersonal, community and health care level. Behavioral theories, such as the theory of planned behavior, were useful for structuring the research and guiding data interpretation. Interventions to support adherence in the region should ideally be directed at all these factors in order for patients to adhere and persist to the treatment, and ultimately for their health and quality of life.

## Competing interests

The authors declare that they have no competing interests.

## Authors’ contributions

RL and DM fashioned the study design, data analyses and interpretation, and drafted the manuscript. MB contributed to study design, data interpretation and analyses, and drafting and reviewing the manuscript. JB, HH and AV contributed in data analysis and interpretation and writing of the article. All authors read and approved the final manuscript.

## Pre-publication history

The pre-publication history for this paper can be accessed here:

http://www.biomedcentral.com/1471-2458/12/716/prepub
